# Draft Genome Sequence of *Aeribacillus pallidus* Strain 8m3, a Thermophilic Hydrocarbon-Oxidizing Bacterium Isolated from the Dagang Oil Field (China)

**DOI:** 10.1128/genomeA.00500-16

**Published:** 2016-06-09

**Authors:** Andrey B. Poltaraus, Diyana S. Sokolova, Denis S. Grouzdev, Timophey M. Ivanov, Sophia G. Malakho, Alena V. Korshunova, Aleksey S. Rozanov, Tatiyana P. Tourova, Tamara N. Nazina

**Affiliations:** a Engelhardt Institute of Molecular Biology, Russian Academy of Sciences, Moscow, Russian Federation; b Research Center of Biotechnology of the Russian Academy of Sciences, Moscow, Russian Federation; c Federal State Budgetary Institution Federal Science Center for Physical Culture and Sport, Moscow, Russian Federation; d Institute of Cytology and Genetics, Siberian Branch of the Russian Academy of Sciences, Novosibirsk, Russian Federation

## Abstract

The draft genome sequence of *Aeribacillus pallidus* strain 8m3, a thermophilic aerobic oil-oxidizing bacterium isolated from production water from the Dagang high-temperature oil field, China, is presented here. The genome is annotated to provide insights into the genomic and phenotypic diversity of the genus *Aeribacillus*.

## GENOME ANNOUNCEMENT

The genus *Aeribacillus* contains a single species, *Aeribacillus pallidus* (formerly *Geobacillus pallidus = Bacillus pallidus*) (1). Representatives of this species have been isolated from sewage (1), production water from high-temperature oilfields (2, 3), hot springs (4, 5), oil-contaminated soil (6), and from a deep geothermal reservoir (7). The only genome of the *A. pallidus* (strain GS3372) deposited at DDBJ/EMBL/GenBank has a G+C content of 57.4% (7). This value is considerably higher than the G+C content of the DNA (39 to 41 mol%) of *A. pallidus* type strain DSM 3670. The strain 8m3 was isolated from a formation water sample from the Dagang high-temperature oil field (Hebei Province, China) (38°40′4.7″N, 117°22′38.0″E) (3). Strain 8m3 is an aerobic endospore-forming bacterium able to grow at temperatures ranging from 38 to 65°C and produces biosurfactants in the course of the degradation of crude oil. According to phylogenetic analysis of the 16S rRNA gene sequence, it was identified as an *A. pallidus* strain (3).

The genomic DNA of this strain was isolated from the biomass using the DNeasy blood and tissue kit (Qiagen, Germany), according to the manufacturer’s instructions. The TruSeq DNA sample preparation kit (Illumina, USA) was used to create the libraries for genome sequencing. Genomic DNA was sequenced using the MiSeq reagent kit version 2 (Illumina). The shotgun library was constructed with a 500-bp paired-end library.

Approximately 7,344,862 paired-end reads were generated, providing 200-fold genome coverage. The resulting reads were assembled with SPAdes version 3.1.0 (8). The remaining 79 contigs were submitted to the ProDeGe website (9) for automatic decontamination. Finally, the assembled sequence was submitted to the National Center for Biotechnology Information (NCBI) Prokaryotic Genome Annotation Pipeline (PGAP) (10) for annotation.

The draft genome sequence of *A. pallidus* strain 8m3 revealed a genome size of 3,818,610 bp, with an average G+C content of 38.86%, which was close to the G+C content of *A. pallidus* type strain DSM 3670. The draft genome contained 3,717 genes, 3,520 coding DNA sequences, 15 coding rRNAs (5S, 16S, and 23S), 60 tRNAs, and 5 noncoding RNAs (ncRNAs). The 16S rRNA and *gyrB* gene sequences revealed in the genome were identical to those determined earlier for the strain (3). Other genes were associated with the degradation of aromatic compounds and fatty acids, utilization of sugars and amino acids, and sporulation. New genomic data were in agreement with the results of phenotypic studies of the strain.

This genome sequence is expected to provide deep insights into the metabolic potential of this organism and its compliance to the conditions of the deep high-temperature petroleum reservoir.

### Nucleotide sequence accession numbers.

This whole-genome shotgun project has been deposited at DDBJ/ENA/GenBank under the accession no. LWBR00000000. The version described in this paper is version LWBR01000000.
